# Very strong correlations of metoprolol- and solanidine-derived metabolic ratios in a real-world cohort with a stable metoprolol drug regimen

**DOI:** 10.3389/fphar.2026.1812007

**Published:** 2026-05-29

**Authors:** Jens Andreas Sarömba, Berkan Kurt, Alessandra Antwerpen, Konstantin Rex, Anna Giacin, Justus Bornemann, Kaan Aygar, Marieke Mertens, Dirk Müller-Wieland, Edgar Dahl, Nikolaus Marx, Florian Kahles, Justyna Wozniak, Jule Neiß, Dominik Krzoska, Julia C. Stingl, Julian Peter Müller, Katja S. Just

**Affiliations:** 1 Institute of Clinical Pharmacology, University Hospital RWTH Aachen, Aachen, Germany; 2 Department of Internal Medicine I - Cardiology, University Hospital Aachen, RWTH Aachen University, Aachen, Germany; 3 RWTH cBMB at the Institute of Pathology, Medical Faculty of RWTH Aachen University, Aachen, Germany; 4 Internal Medicine IX – Department of Clinical Pharmacology and Pharmacoepidemiology, Medical Faculty Heidelberg/Heidelberg University Hospital, Heidelberg University, Heidelberg, Germany

**Keywords:** biomarkers, CYP2D6, liquid chromatography–mass spectrometry, metoprolol, pharmacogenomics, solanidine

## Abstract

**Introduction:**

Individual CYP2D6 activity can be assessed with minimal invasiveness by genotyping and solanidine-derived phenotyping. In this study, we aimed to compare the two methods to predict the metabolism of the CYP2D6 substrate metoprolol in a cohort of the “All-comer” Registry for ImmunocArdiology aNd cardiometabolic disease Aachen (ARIANA) study (DRKS00025716; https://drks.de/search/de/trial/DRKS00025716) with a stable metoprolol regimen.

**Methods:**

In total, N = 48 patients were clinically assessed, and their medical records were analyzed for CYP2D6-interfering drugs. Plasma samples collected in a fasted state were analyzed using liquid chromatography–tandem mass spectrometry (LC–MS). Genotyping was performed using the Infinium Global Diversity Array with Enhanced PGx. CYP2D6 activity scores and metabolic ratios (MRs) of solanidine and metoprolol were calculated.

**Results:**

N = 47 patients with detectable metoprolol plasma concentrations were included in the primary analysis. The MRs ln MR (OH-solanidine/solanidine) and ln MR (3,4-seco solanidine-3,4-dioic acid/solanidine) correlated very strongly (r = 0.907 and r = 0.945) with the ln MR (OH-metoprolol/metoprolol). There was a strong negative correlation of both solanidine-derived MRs with metoprolol trough levels (r = −0.625 and r = −0.703). The genotyping-derived CYP2D6 activity score showed weaker correlations in all comparisons, even when adjusting for covariates.

**Discussion:**

Solanidine-derived phenotyping showed stronger correlations to the measured CYP2D6 activity and metoprolol trough levels than the genotyping-derived activity scores. More research is needed in order to implement solanidine-derived dosing recommendations into clinical practice.

## Introduction

Precision medicine aims to deliver the right drug at the right dose to each individual patient, thereby optimizing efficacy while minimizing the risk of adverse drug reactions (ADRs) (Verstegen and Ito, 2019). A particular focus of research (Nofziger et al., 2020; Taylor et al., 2020; Aurinsalo et al., 2025b) and clinical implementation (Størset et al., 2025; Swen et al., 2023; Turner et al., 2023) has been on drugs metabolized by cytochrome P450 2D6 (CYP2D6). This enzyme is responsible for the metabolism of the majority of psychoactive drugs and around a quarter of all drugs prescribed (Owen et al., 2009). At the same time, it is highly polymorphic, showing a marked inter- and intrapopulation variance that may facilitate unwanted drug responses in ethnic minorities and patients with either elevated or reduced CYP2D6 activity (Kehinde et al., 2023).

Metoprolol is a beta-adrenoceptor antagonist mostly used in a wide range of cardiovascular conditions, making it the fifth most prescribed drug in the United States (Fournier et al., 2024). It is predominantly metabolized by CYP2D6 (McGourty et al., 1985), a property which is exploited in pharmacokinetic studies (Bachmann et al., 2021; Paludetto et al., 2023). The Clinical Pharmacogenetics Implementation Consortium (CPIC) released a guideline (Duarte et al., 2024) for patients with available genotyping results recommending that patients encoding no functional CYP2D6 enzymes lower their first metoprolol dose by half. No further recommendations were given for patients with otherwise reduced or elevated CYP2D6 activity scores, citing a lack of clinical evidence.

Historically, phenotyping of drug metabolism-facilitating enzymes with specific probe drugs has been viewed as an alternative approach to tailor therapy according to individual enzymatic capacity. Advantages cited included fast and easy-to-interpret results and incorporation of environmental factors, that is, enzyme induction and inhibition, into the readout (Opdam et al., 2015). However, due to various logistical, medical, and regulatory hurdles, incorporating probe drug administration into clinical practice has proven to be difficult, which partly explains the dominant role genotyping has in precision medicine today (Gaedigk et al., 1999; McElroy et al., 2000; Frank et al., 2007).

Measuring solanidine and its metabolites in plasma has been proposed as a minimally invasive way to phenotype CYP2D6 without prior administration of a probe drug (Aurinsalo et al., 2025a; Magliocco et al., 2021; Kiiski et al., 2024; Medwid et al., 2024; Sarömba et al., 2025). Muller et al. (2024) showed correlations of solanidine and metoprolol metabolic ratios (MRs) in volunteers after administering a single dose of 12.5 mg oral metoprolol during a cocktail study. However, the translation of solanidine-derived phenotyping into clinical practice is lacking.

In this study, we aimed to assess the predictive performance of solanidine-derived phenotyping of CYP2D6 using MRs of solanidine in patients on stable metoprolol therapy and compare it to genotyping.

## Materials and methods

### Study design

The analyzed cohort is a subset of the ongoing “All-comer” Registry for ImmunocArdiology aNd cardiometabolic disease Aachen (ARIANA; study ID: DRKS00025716, registered on 30 August 2021), which enrolls patients who were hospitalized at the cardiology department of the University Hospital Aachen for various medical reasons. Fasted patients aged 50 or older were eligible for inclusion when no external factors promoting systemic inflammation were apparent.

Our subset comprises enrolled patients with both a documented stable intake of metoprolol for at least 1 week, regardless of the indication for which it was initially prescribed, and an available blood sample before the next intake of metoprolol (blood trough level). Patients taking metoprolol in two equal doses in the morning and the evening, with a daily dose of either 95 mg or 190 mg, were eligible for inclusion in our study. Adherence to this medication was assessed after enrollment by measuring metoprolol in plasma. The sample size of the subset was calculated as follows: in order to detect a correlation between ln MR OH-metoprolol/metoprolol and ln MR SSDA/solanidine with r = 0.596 (Muller et al., 2024), alpha = 0.05, and 90% power, N = 25 patients must be enrolled. To allow for a lack of compliance and the effect of comorbidities, N = 48 patients were enrolled. All participants provided written informed consent, and the study was approved by the Internal Ethics Review Board at University Hospital RWTH Aachen (EK 234/21). This study was carried out in concordance with the declaration of Helsinki and all at-the-time current amendments (Association, 2013; 2025) thereof.

### Clinical assessment

Vital signs (blood pressure and heart rate) were retrieved from electronic medical records, using the measurements recorded closest in time to the blood draw. Information on demographics, anthropometrics, cardiovascular (CV) risk factors, medical conditions, comorbidities, and medication was received from medical records and physician-validated discharge letters of all assessed patients. Glomerular filtration rate was estimated based on creatinine measurements (Inker et al., 2021). Pack years of tobacco use were calculated. Drug intake was fully documented for all patients in the subset. Patients’ medical records were analyzed for documented CYP2D6 inhibitor use according to the Flockhart table (Flockhart Da, 2021).

### Acquisition and handling of patient samples

Blood was drawn before drug intake into tubes containing ethylene diamine tetra-acetic acid (EDTA). Samples were either temporarily refrigerated at 5 °C for up to 6 h or immediately transferred to the biobanking facilities for further processing. Whole blood was aliquoted and stored at −80 °C. For plasma, EDTA blood was centrifuged at 2,500 g for 10 min. Plasma was then aliquoted and stored at −80 °C until further analysis.

### LC-MS analysis

The following materials were used for the liquid chromatography–mass spectrometry (LC–MS) methods: solanidine (13264-1MG; Sigma), formic acid (84865.180, HiPerSolv; VWR Chemicals), metoprolol (HY-17503; MedChemExpress), α-OH-metoprolol (Cay28020-1; Biomol), methanol (ultra-gradient HPLC-grade; 8402; J.T. Baker), water (LiChrosolv, liquid chromatography–mass spectrometry (LC-MS)-grade, 1.15333.2500; Merck), metoprolol-d6 (Cay28188-1; Biomol), α-OH-metoprolol-d5 (TOR-H948392-1MG; Biozol), dextromethorphan-d3 solution, 100 µg/mL (D-071-1ML; Sigma Aldrich), and bovine serum (2203-010; Acila). The synthesis of 4-hydroxy-solanidine, referred to as OH-solanidine in this manuscript, and the preparation of charcoal stripped plasma have been described previously (Sarömba et al., 2025).

Levels of metoprolol, solanidine, and the respective metabolites were measured with two LC–MS methods. Both used the Agilent 1290 Infinity II ultra-high performance liquid chromatography system with a Poroshell 120 EC-C18 column (1.9 μm, 2.1 mm × 50 mm, 699675-902, Agilent Technologies) coupled to a SCIEX QTRAP6500+ mass spectrometer. Mobile phase A was 0.1% (v/v) formic acid in water, and mobile phase B was methanol.

Metoprolol and OH-metoprolol were quantified by a validated method reported previously with minor modifications (Muller et al., 2024; Müller et al., 2025). In short, 80 μL of methanol with internal standards metoprolol-d6 and OH-metoprolol-d5 was added to 20 μL plasma and vortexed for 10 s. After centrifuging at 17,000 g at 4 °C for 20 min, 40 μL of supernatant was diluted with 40 μL water and centrifuged again for 5 min at 17,000 g at 4 °C, and the supernatant was transferred to an HPLC vial. The injection volume was 5 μL, and the flow rate was 0.7 mL/min. Metoprolol and OH-metoprolol were quantified in a linear range from 0.25 ng/mL to 200 ng/mL. Samples exceeding levels of 200 ng/mL were diluted 1:10 with bovine serum and reanalyzed.

Solanidine and its metabolites, OH-solanidine and 3,4-secosolanidine-3,4-dioic acid (SSDA), were quantified using a modified method published previously (Sarömba et al., 2025). A 50-μL aliquot of plasma was protein precipitated with 200 μL methanol containing 0.1% (v/v) formic acid and 1 ng/mL dextromethorphan-d3 as a load control and vortexed for 10 s. The first centrifugation was at 17,000 g at 4 °C for 20 min, then 120 μL supernatant was centrifuged at 17,000 g at 4 °C for 10 min. Finally, 100 μL supernatant was transferred into an HPLC vial. The injection volume was 14 μL, and the flow rate was 0.7 mL/min. Details of the LC–MS methods used and measures of accuracy and precision can be found in Supplementary Material Sl. SSDA was unavailable as a standard. Therefore, the assessment is semi-quantitative.

### Genotyping

Genotyping was performed by Life and Brain GmbH, Bonn, Germany, using the Illumina Global Diversity Array (GDA) assay with enhanced pharmacogenetics. A list of assessed single-nucleotide polymorphisms (SNPs) for CYP2D6 can be found in Supplementary Material S2. Genotypes were retrieved using STARGAZER_V2.0 (Lee et al., 2019). CYP2D6 copy number variation was analyzed using Illumina’s Dynamic Read Analysis for GENomics (DRAGEN) Array v1.1. CYP2D6 activity scores were calculated using CPIC resources (PharmGKB, 2024). In order to calculate the activity score (AS) of genotype-predicted undetermined metabolizers, rare variants were assigned an AS of 1. The Hardy–Weinberg equilibria were assessed for each rs number.

### Statistical analysis

For patient characteristics of the population, absolute numbers and percentages were calculated for the categorical variables. Medians and interquartile ranges were calculated for continuous variables. To assess the CYP2D6-mediated metabolism of metoprolol, we first examined the natural logarithm of the metabolic ratio ln MR (OH-metoprolol/metoprolol) as it is a widely accepted measure of CYP2D6 activity. In the second step, we correlated the trough levels of metoprolol as they are predictors of treatment efficacy in univariate analysis. Both univariate relationships were analyzed using correlation and linear regression of solanidine with metoprolol MRs and metoprolol trough levels, respectively, each with a different measure of CYP2D6 activity: lnMR (OH-solanidine/solanidine), lnMR (SSDA/solanidine), lnMR (OH-solanidine × SSDA/solanidine^2^), and the CYP2D6 AS. In order to incorporate the known clinical and demographic determinants of metoprolol metabolism, we attempted a linear multiple regression analysis of the metoprolol trough levels as dependent variables. The following independent variables were used: Age was included as continuous, and male sex, metoprolol dose (daily 95 mg or 190 mg), and CYP2D6 inhibitor intake (yes vs. no) were treated as binary variables. We also attempted to replicate the known correlation (Muller et al., 2024; Sarömba et al., 2025) between both the lnMR (OH-solanidine/solanidine) and lnMR (SSDA/solanidine) and the CYP2D6 AS. We hypothesize that a measure of CYP2D6 activity, which incorporates three inputs, may be more robust than metabolic ratios and may therefore be a better predictor of metoprolol metabolism. In an exploratory analysis, we attempted to measure the performance of the sum of the two used solanidine-derived metabolic ratios, that is, ln (SSDA × OH-solanidine/solanidine^2^). Point estimates and corresponding 95% confidence intervals (CI) were calculated for all variables. Correlation coefficients are described as follows: r < 0.200, very weak; r = 0.200–0.399, weak; r = 0.400–0.599, moderate; r = 0.600–0.799, strong; and r > 0.800, very strong (Evans, 1996). All statistical analyses were performed using Graphpad Prism 10.0, and *p*-values < 0.05 were considered statistically significant.

## Results

A total of 48 patients with documented stable metoprolol use were included in this study. Metoprolol and OH-metoprolol were undetectable in one patient, who was excluded from further analysis, leaving a total of N = 47 patients analyzed. Thorough analysis of the excluded patient’s medication history revealed discontinuation of metoprolol 3 days before sampling. All 47 patients took metoprolol in two equal doses in the morning and the evening, with 23 patients taking a daily dose of 95 mg and 24 patients taking a daily dose of 190 mg. Patient characteristics are reported in Table 1. Patients were, in median, 75 years old (IQR 69; 81) and took, in median, seven drugs (IQR: 5; 11). Genotyping results for CYP2D6 with predicted phenotypes are reported in Table 2 with all alleles being in Hardy–Weinberg equilibrium. Nine patients took a drug classified as a CYP2D6 inhibitor, with amiodarone being the CYP2D6 inhibitor taken most often (Supplementary Material S3).

**TABLE 1:** Patient characteristics of a subset of the ARIANA (“All-comer” Registry for ImmunocArdiology aNd cardiometabolic disease Aachen) study comprising patients with detected metoprolol levels (N = 47).

Characteristic	Missing value	Characteristic
Age (years), median (IQR)	-	75 (69; 81)
Male sex, n (%)	-	28 (59.6)
Body height (cm), median (IQR)	-	173 (165; 178)
Body weight (kg), median (IQR)	-	83.5 (74.2; 94.7)
Body mass index, median (IQR)	-	27.6 (25.2; 30.6)
Caucasian descent, n (%)	-	46 (97.9)
North African descent, n (%)	-	1 (2.1)
Systolic blood pressure (mm Hg), median (IQR)	-	125 (110; 147)
Diastolic blood pressure (mm Hg), median (IQR)	-	74 (68; 80)
Heart rate (beats per minute), median (IQR)	-	64 (60; 75)
Dyslipidemia or intake of lipid-lowering medications, n (%)	-	41 (87.2)
Type 2 diabetes mellitus, n (%)	-	13 (27.7)
Currently smoking tobacco or using other nicotine-containing products, n (%)	-	3 (6.4)
Pack years of cigarette smoking, median (IQR)	3	6 (0; 30)
Alcohol dependency, n (%)	-	1 (2.1)
Coronary artery disease, n (%)	-	31 (70.0)
Atherosclerotic cardiovascular disease, n (%)	-	35 (74.5)
Heart failure with reduced ejection fraction, n (%)	-	22 (46.8)
Heart failure with preserved ejection fraction, n (%)	-	11 (23.4)
Aortic stenosis, n (%)	-	9 (19.1)
Atrial fibrillation, n (%)	-	29 (61.7)
Depression, n (%)	-	2 (4.3)
Chronic obstructive pulmonary disorder, n (%)	-	10 (21.3)
Chronic kidney disease, n (%)	-	14 (29.8)
Estimated creatinine-based glomerular filtration rate (ml/min/1.73 m^2^), median (IQR)	-	65.7 (48.8; 77.6)
Number of medications, median (IQR)	-	7 (5; 11)
Aspartate transaminase (U/I)	-	23 (19; 30.5)
Alanine aminotransferase (U/I)	-	20 (16; 30)

IQR: interquartile range.

**TABLE 2:** Diplotypes of cytochrome P450 (CYP) 2D6 and respective CYP2D6 activity scores and genotype-predicted phenotypes in a subset of the ARIANA (“All-comer” Registry for ImmunocArdiology aNd cardiometabolic disease Aachen) study comprising patients with detected metoprolol levels (N = 47).

Predicted phenotype	Assigned activity score	Diplotype	Frequency, n (%)
Poor metabolizer (PM)	0	*4/*4	2 (4.3)
Intermediate metabolizer (IM)	0.25	*4/*41	3 (6.4)
		*5/*41	1 (2.1)
	1	*1/*3	1 (2.1)
		*1/*4	11 (23.4)
		*1/*5	2 (4.3)
		*1/*6	1 (2.1)
		*2/*4	1 (2.1)
		*2/*5	1 (2.1)
		*5/*33	1 (2.1)
		*6/*33	1 (2.1)
Normal metabolizer (NM)	1.25	*1/*9	1 (2.1)
		*1/*10	1 (2.1)
		*2/*9	2 (4.3)
		*2/*41	2 (4.3)
	2	*1/*1	4 (8.5)
		*1/*2	2 (4.3)
		*1/*35	3 (6.4)
		*2/*2	2 (4.3)
		*35/*35	1 (2.1)
Ultra-rapid metabolizer (UM)	3	*1/*2 × 2	1 (2.1)
Undetermined metabolizer (XM)	1	*4/*23	1 (2.1)
	1.25	*22/*41	1 (2.1)
	2	*1/*108	1 (2.1)

**FIGURE 1:**
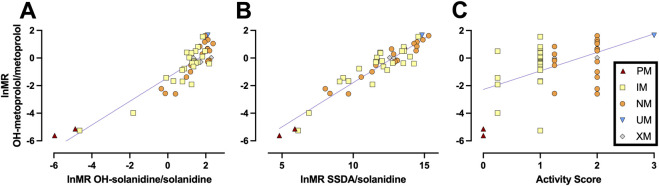
Linear regression of the lnMR (OH-metoprolol/metoprolol) in a subset of the ARIANA (“All-comer” Registry for ImmunocArdiology aNd cardiometabolic disease Aachen) study comprising patients with detected metoprolol levels (N = 47) with **(A)** lnMR (OH-solanidine/solanidine): r = 0.907 (95 CI: 0.838–0.948) and R^2^ = 0.823 (*p* < 0.0001), **(B)** lnMR (SSDA/solanidine): r = 0.946 (95 CI: 0.905–0.970) and R^2^ = 0.896 (*p* < 0.0001), and **(C)** CYP2D6 activity score as predicted by genotype: r = 0.500 (95 CI: 0.249–0.688) and R^2^ = 0.250 (*p* = 0.0003). Ln, natural logarithm; MR, metabolic ratio; SSDA, 3,4-secosolanidine-3,4-dioic acid; PM, genotype-predicted poor metabolizer; IM, genotype-predicted intermediate metabolizer; NM, genotype-predicted normal metabolizer; UM, genotype-predicted ultra-rapid metabolizer; XM, genotype-predicted indeterminate metabolizer; 95 CI, 95% confidence interval; CYP2D6, cytochrome P450 2D6.

Solanidine or its metabolites were detected in all 47 patients. The metabolic ratio lnMR (OH-metoprolol/metoprolol) correlated very strongly with the lnMR (OH-solanidine/solanidine), yielding an r = 0.907 (95 CI: 0.838–0.948) and R^2^ = 0.823 (Figure 1A), and with the lnMR (SSDA/solanidine), yielding an r = 0.946 (95 CI: 0.905–0.970) and R^2^ = 0.896 (Figure 1B). The lnMR (OH-metoprolol/metoprolol) correlated moderately with the CYP2D6 AS, yielding an r = 0.500 (95 CI: 0.249–0.688) and R^2^ = 0.250 (Figure 1C). Interestingly, we found numerically stronger correlations for the sum of the two solanidine MRs (Supplementary Materials S4, S5).

**FIGURE 2:**
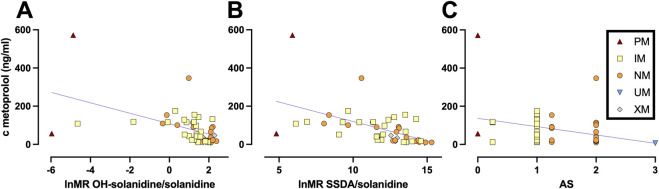
Linear regression of metoprolol trough levels in a subset of the ARIANA (“All-comer” Registry for ImmunocArdiology aNd cardiometabolic disease Aachen) study comprising patients with detected metoprolol levels (N = 47) with **(A)** lnMR (OH-solanidine/solanidine): r = −0.513 (95 CI: −0.698 to −0.265) and R^2^ = 0.263 (*p* < 0.0002), **(B)** lnMR (SSDA/solanidine): r = −0.533 (95 CI: −0.711 to −0.291) and R^2^ = 0.284 (*p* = 0.0001), and **(C)** CYP2D6 activity score: r = −0.288 (95 CI: −0.531 to −0.001) and R^2^ = 0.083 (*p* = 0.499). Ln, natural logarithm; MR, metabolic ratio; SSDA, 3,4-secosolanidine 3,4-dioic acid; PM, genotype-predicted poor metabolizer; IM, genotype-predicted intermediate metabolizer; NM, genotype-predicted normal metabolizer; UM, genotype-predicted ultra-rapid metabolizer; XM, genotype-predicted indeterminate metabolizer; 95 CI, 95% confidence interval; CYP2D6, cytochrome P450 2D6.

When correlating metoprolol trough levels, we found moderate negative correlations with lnMR (OH-solanidine/solanidine) (r = −0.513 (95 CI: −0.698 to −0.265), R^2^ = 0.263) (Figure 2A), moderate negative correlations with lnMR (SSDA/solanidine) (r = −0.533 (95 CI: 0.711 to −0.291), R^2^ = 0.284) (Figure 2B), and weak negative correlation with the CYP2D6 AS (r = −0.288 (95 CI: −0.531 to −0.001), R^2^ = 0.083) (Figure 2C).

In a multiple regression model incorporating age, sex, CYP2D6 inhibitor intake, and daily dose of metoprolol, in addition to different measures of CYP2D6 activity, with metoprolol plasma concentration as the dependent variable, all three measures of CYP2D6 activity were significantly associated. The model basing CYP2D6 activity on lnMR (OH-solanidine/solanidine) yielded a R^2^ = 0.614, the model based on lnMR (SSDA/solanidine) yielded a R^2^ = 0.599, and the model based on the CYP2D6 AS yielded a R^2^ = 0.386 (Table 3). There were significant associations with all measures of CYP2D6 activity and age but not with sex and CYP2D6 inhibitor intake.

**TABLE 3:** Linear multiple regression of different measures of CYP2D6 activity in a subset of the ARIANA (“All-comer” Registry for ImmunocArdiology aNd cardiometabolic disease Aachen) study comprising patients with detected metoprolol levels (N = 47) with metoprolol trough levels as the dependent variable.

Variable	Model 1 CYP2D6 activity by lnMR (OH-solanidine/Solanidine), beta estimate (95% CI) R^2^ = 0.614	Model 2 CYP2D6 activity by lnMR (SSDA/Solanidine), beta estimate (95% CI) R^2^ = 0.599	Model 3 CYP2D6 activity by genotype-predicted activity score, beta estimate (95% CI) R^2^ = 0.386
Intercept	−78.8 (−251.3 to 93.7)	122 (−80.0 to 324.0)	−86.1 (−309.3 to 137.0)
Measure of CYP2D6 activity	**−30.0 (−40.9 to −19.2)**	**−21.7 (−29.9 to −13.5)**	**−41.0 (−80.4 to −1.5)**
Age (years)	**2.5 (0.3 to 4.7)**	**2.7 (0.5 to 4.9)**	**2.9 (0.2 to 5.7)**
Male sex	−59.3 (−3.5 to 94.0)	−39.9 (−83.1 to 3.4)	**−53.3 (−106.6 to −0.1)**
CYP2D6 inhibitor intake	45.3 (−3.5 to 94.0)	25.8 (−24.0 to 75.7)	39.2 (−22.2 to 100.6)
Daily dose of 190 mg metoprolol	**62.7 (22.4 to 103.0)**	**72.5 (30.3 to 114.7)**	50.5 (−0.7 to 101.8)

CI, confidence interval; CYP2D6, cytochrome P450 (CYP) 2D6. Findings with *p* < 0.05 are shown in bold text.

## Discussion

In this study, we found significant correlations between the four measurements of CYP2D6 activity, namely, the CYP2D6 activity score (AS), lnMR (OH-metoprolol/metoprolol), lnMR (OH-solanidine/solanidine), and lnMR (SSDA/solanidine). We found stronger negative correlations between the solanidine-derived MRs and the metoprolol plasma concentrations than with CYP2D6 genotyping. When adjusting for covariates, we also found better-fitting models based on the solanidine-derived MRs than with the genotype-predicted CYP2D6 AS.

Our results show, that solanidine-derived phenotyping correlates better with the actual CYP2D6 activity than the AS most likely due to a combination of genetic and non-genetic influences: While a twin study by Matthaei et al. (2015) estimated 91% of the pharmacokinetic variation of metoprolol to be due to additive genetic effects, the variability in the CYP2D6 gene, assessed with an AS-based approach, explains less than half of that (Ning et al., 2018; Dalton et al., 2020), suggesting contributions of both distant genes and unassessed parts of the CYP2D6 locus. Interpretation of the complex genetic basis of inherited CYP2D6 activity is avoided when performing solanidine-derived phenotyping as the MRs are read out directly, which is a strength of the method. However, efforts to broaden the scope of genotyping, such as pharmacoepigenomics (Griñán-Ferré et al., 2024), show promise in improving the resolution of pharmacogenetic results. Environmental influences on CYP2D6 activity, such as CYP2D6 inhibition by comedications, the impact of supplements and nutrition through plant secondary metabolites, or altered body function by comorbidities and lifestyle choices, are also factored indirectly into the phenotyping readout, whereas they can only be estimated when incorporating a genotyping-based approach (Opdam et al., 2015; Sarömba et al., 2025).

This interpretation is in line with a work by Wollmann et al. (2024), who found strong correlations of the lnMR (9-OH-risperidone/risperidone) and lnMR (SSDA/solanidine) when analyzing psychiatric patients with an AS of 1 and 1.5, respectively, indicating significant inter-patient variability in CYP2D6 activity irrespective of the AS. On the other hand, when predicting endoxifen concentrations above or below the threshold of 16 nM in breast cancer patients undergoing tamoxifen therapy, Medwid et al. (2024) found that a model incorporating genotype-predicted CYP2D6 phenotypes and antidepressant inhibitor use performed better numerically than models based on solanidine-derived phenotyping (Medwid et al., 2024).

Advantages of solanidine-derived phenotyping include the very strong correlation between measured CYP2D6 activity and the moderate (Borges et al., 2010) to strong (Muller et al., 2024) correlations of the genotyping-derived AS. Furthermore, results from solanidine-derived phenotyping can be obtained in several hours, whereas genotyping usually takes several days. Another strength is the direct assessment of phenoconversion (Sarömba et al., 2025). Finally, solanidine-derived phenotyping can be implemented into existing therapeutic drug monitoring programs with relative ease (Størset et al., 2025). Disadvantages include the variability of the results obtained over time, which may make repeated testing necessary (Einvik, 2023; Muller et al., 2024). Moreover, no clinical guidelines incorporating CYP2D6 are available. Finally, there is no reimbursement for solanidine-derived phenotyping. However, reimbursement for genotyping is likewise differently organized and lacking in many places worldwide (Abou Diwan et al., 2019).

Strengths of this study include the broad panel of assessed SNPs of the CYP2D6 gene, enabling us to detect rare variants of the gene. In addition, this analysis is based on a subsample of a real-world study characterized by rigorous clinical data curation and physician validation as all information was derived from medical reports. Consequently, only physician-confirmed, guideline-concordant diagnoses were included, rather than registry-based or health claims data. Our older, multimorbid cohort presented with a diverse spectrum of cardiological diagnoses requiring metoprolol therapy, which in our view represents the variety of typical patients in clinical practice and reflects the use of metoprolol for several cardiological diagnoses. As the application of solanidine-derived phenotyping has been demonstrated in psychiatric (Wollmann et al., 2024), breast cancer (Medwid et al., 2024), and geriatric (Sarömba et al., 2025) patients, it is likely feasible in most clinical populations.

Limitations of this study include a rather small sample size and unsupervised dosing of metoprolol. Nevertheless, we found moderate correlations between solanidine-derived MRs and plasma trough levels. Our genotyping method did not test for NFIB rs28379954 T>C polymorphism, which arguably affects CYP2D6 activity measured by solanidine-derived phenotyping (Smith et al., 2024; Medwid et al., 1990), and we did not discern structural variants such as CYP2D6/CYP2D7 hybrid genes. However, due to the rarity of these variants and given the relatively small sample size, only a low impact on the study results would be expected.

In conclusion, we found strong correlations between solanidine-derived MRs and the validated CYP2D6 activity marker lnMR (OH-metoprolol/metoprolol) as well as metoprolol trough levels. The correlations with the CYP2D6 AS were weaker, even when adjusting for covariates. These findings indicate that solanidine MRs are stronger predictors of metoprolol metabolism and CYP2D6 activity than the CYP2D6 genotype. We advocate for more research to validate solanidine-derived phenotyping for dosage recommendations of metoprolol and other CYP2D6 substrates, aiming for implementation into clinical practice.

## Data Availability

Original data were uploaded to a repository accessible under the following link: https://github.com/katjajust/Solandine-Metoprolol-Correlations.
