# Supplementary material for: Very strong correlations of metoprolol- and solanidine-derived metabolic ratios in a real-world cohort with a stable metoprolol drug regimen
Source: Front Pharmacol. 2026 May 29;17:1812007. doi: 10.3389/fphar.2026.1812007 (PMC13260174; doi:10.3389/fphar.2026.1812007)
Supplement: Supplementary file 1 [file Supplementaryfile1.docx]

## Supplement 1

**Supplement 1**: LC-MS methods, accuracy and precision of the LC-MS methods.

**Table S1.1**: Gradient of the method used to quantify metoprolol and OH-metoprolol.

| **Time (min)** | **%B** |
| --- | --- |
| 0 | 5 |
| 0.2 | 5 |
| 2 | 30 |
| 4.8 | 34 |
| 6.5 | 95 |
| 7.5 | 95 |
| 7.6 | 5 |
| 8.8 | 5 |

**Table S1.2**: MS transitions of the method used to quantify metoprolol and OH-metoprolol.

| Molecule | Status | Q1 mass (Da) | Q3 mass (Da) | Expected retention time (min) | Declustering potential (V) | Collision energy (V) | Collision cell exit potential |
| --- | --- | --- | --- | --- | --- | --- | --- |
| Metoprolol | Quantifier | 268.01 | 116.00 | 2.67 | 46.00 | 25.00 | 14.00 |
| Metoprolol | Qualifier | 268.01 | 121.00 | 2.67 | 46.00 | 31.00 | 18.00 |
| OH-metoprolol | Quantifier | 284.13 | 116.10 | 1.86 | 66.00 | 25.00 | 18.00 |
| OH-metoprolol | Qualifier | 284.13 | 56.10 | 1.86 | 66.00 | 55.00 | 24.00 |

**Table S.1.3**: Gradient of the HPLC method used to quantify solanidine, OH-solanidine and 3,4, seco solanidine 3,4 dioic acid.

| **Time (min)** | **%B** |
| --- | --- |
| 0 | 5 |
| 0.10 | 5 |
| 0.15 | 37 |
| 0.90 | 37 |
| 1.40 | 53 |
| 1.65 | 53 |
| 1.85 | 59.3 |
| 1.95 | 70 |
| 2.1 | 70 |
| 2.4 | 99 |
| 3.9 | 99 |
| 4.0 | 96.5 |
| 4.65 | 96.5 |
| 4.7 | 5 |
| 5 | 5 |

**Table S1.4**: MS transitions of the method used to quantify solanidine, OH-solanidine and 3,4, seco solanidine 3,4 dioic acid (SSDA).

| Molecule | Status | Q1 mass (Da) | Q3 mass (Da) | Expected retention time (min) | Declustering potential (V) | Collision energy (V) | Collision cell exit potential |
| --- | --- | --- | --- | --- | --- | --- | --- |
| Dextromethorphan-d3 | Load control | 275.20 | 215.20 | 2.1 | 96.0 | 41.0 | 12.0 |
| SSDA | Quantifier | 444.30 | 370.30 | 2.15 | 50.0 | 55.0 | 12.0 |
| SSDA | Qualifier | 444.30 | 98.10 | 2.15 | 50.0 | 65.0 | 12.0 |
| OH-solanidine | Quantifier | 414.30 | 98.10 | 2.50 | 50.0 | 65.0 | 12.0 |
| OH-solanidine | Qualifier | 414.30 | 398.30 | 2.50 | 50.0 | 63.0 | 12.0 |
| Solanidine | Quantifier | 398.30 | 98.10 | 2.57 | 50.0 | 55.0 | 14.0 |
| Solanidine | Qualifier | 398.30 | 382.30 | 2.57 | 50.0 | 63.0 | 20.0 |

**Table S1.5**: Calibrators and quality controls for solanidine.

| Concentration (ng/ml) | Status | Number of values | Coefficient of variation (%) | Average accuracy (%) |
| --- | --- | --- | --- | --- |
| 0.01 | Calibrator | 1 | N/A | 93.9 |
| 0.02 | Calibrator | 2 | 15.9 | 102.5 |
| 0.03 | Calibrator | 2 | 5.5 | 110.1 |
| 0.06 | Calibrator | 2 | 1.5 | 89.4 |
| 0.20 | Calibrator | 2 | 0.6 | 101.2 |
| 1 | Calibrator | 2 | 2.7 | 110.1 |
| 5 | Calibrator | 2 | 5.9 | 99.2 |
| 10 | Calibrator | 2 | 8.4 | 89.9 |
| 0.01 | Quality Control | 2 | 2 | 102.5 |
| 0.02 | Quality Control | 1 | N/A | 91.2 |
| 0.04 | Quality Control | 2 | 2.2 | 87.8 |
| 4.5 | Quality Control | 2 | 7.4 | 91.2 |
| 9 | Quality Control | 2 | 1.2 | 95.9 |

**Table S1.6**: Calibrators and Quality Controls for OH-solanidine.

| Concentration (ng/ml) | Status | Number of values | Coefficient of variation (%) | Average accuracy (%) |
| --- | --- | --- | --- | --- |
| 0.01 | Calibrator | 1 | N/A | 100.5 |
| 0.02 | Calibrator | 2 | 6.1 | 98.3 |
| 0.03 | Calibrator | 2 | 0.0 | 97.6 |
| 0.06 | Calibrator | 2 | 0.5 | 90.6 |
| 0.20 | Calibrator | 2 | 0.2 | 105.2 |
| 1 | Calibrator | 2 | 0.9 | 108.2 |
| 5 | Calibrator | 2 | 2.7 | 101.7 |
| 10 | Calibrator | 2 | 2.6 | 98.3 |
| 0.01 | Quality Control | 2 | 8.0 | 90.9 |
| 0.02 | Quality Control | 2 | 5.1 | 95.4 |
| 0.04 | Quality Control | 2 | 1.7 | 94.5 |
| 4.5 | Quality Control | 2 | 7.2 | 87.5 |
| 9 | Quality Control | 2 | 10.0 | 93.0 |

**Table S1.7**: Calibrators and quality controls for metoprolol.

| Concentration (ng/ml) | Status | Number of values | Coefficient of variation (%) | Average accuracy (%) |
| --- | --- | --- | --- | --- |
| 0.25 | Calibrator | 2 | 12.9 | 97.1 |
| 0.5 | Calibrator | 2 | 0.1 | 107.8 |
| 2 | Calibrator | 2 | 5.0 | 91.5 |
| 10 | Calibrator | 2 | 2.6 | 105.6 |
| 100 | Calibrator | 2 | 0.9 | 97.0 |
| 200 | Calibrator | 2 | 3.5 | 101.1 |
| 0.25 | Quality Control | 2 | 0.9 | 86.8 |
| 0.5 | Quality Control | 2 | 7.0 | 97.6 |
| 60 | Quality Control | 2 | 0.1 | 106.8 |
| 150 | Quality Control | 2 | 0.2 | 108.5 |

**Table S1.8**: Calibrators and quality controls for OH-metoprolol.

| Concentration (ng/ml) | Status | Number of values | Coefficient of variation (%) | Average accuracy (%) |
| --- | --- | --- | --- | --- |
| 0.25 | Calibrator | 2 | 10.9 | 95.5 |
| 0.5 | Calibrator | 2 | 12.5 | 109.5 |
| 2 | Calibrator | 2 | 3.0 | 96.9 |
| 10 | Calibrator | 2 | 0.8 | 106.8 |
| 100 | Calibrator | 2 | 1.0 | 96.5 |
| 200 | Calibrator | 2 | 0.2 | 95.8 |
| 0.25 | Quality Control | 2 | 9.1 | 118.2 |
| 0.5 | Quality Control | 2 | 4.8 | 113.4 |
| 60 | Quality Control | 2 | 2.7 | 92.7 |
| 150 | Quality Control | 2 | 1.4 | 90.9 |

## Supplement 2

**Supplement 2**: Assessed single nucleotide polymorphism for cytochrome P450 2D6 in a subset of the ARIANA-study („All-comer“ Registry for ImmunocArdiology aNd cardiometabolic disease Aachen) comprising patients with detected metoprolol levels (N=47).

| **rs number** | **Associated star alleles** |
| --- | --- |
| rs773790593 | *87 |
| rs72549358 | *28 |
| rs769258 | *35,*143,*172,*175 |
| rs267608313 | *47,*143, |
| rs28371696 | *15,*43,*46,146,*148 |
| rs138100349 | *22,*44,*142, |
| rs1065852 | *4,*10,*36,*37,*47,*49,*52,*54,*56,*64,*65,*69,*72,*87,*94,*95,*99,*100,*101,*114,*132,*142,*147,*150,*177 |
| rs5030862 | *12 |
| rs118203758 | *71,*168 |
| rs774671100 | *15 |
| rs201377835 | *11 |
| rs267608310 | *23 |
| rs267608276 | *99 |
| rs267608309 | *48,*102,*103 |
| rs28371703 | *4,*74,*82,*160 |
| rs28371704 | *4,*82,*160 |
| rs76802407 | *149 |
| rs267608308 | *73 |
| rs76187628 | *82,*88 |
| rs28371706 | *17,*40,*58,*64,*82,*141,*154 |
| rs78459009 | *82 |
| rs535642512 | *111 |
| rs374616348 | *70,*161,*171 |
| rs1135822 | *36,*49,*53, |
| rs1135823 | *36,*53 |
| rs781457579 | *126 |
| rs61736512 | *29,*70,*107,*149,*155,*156,*157,*164,*165,*171 |
| rs375135093 | *89 |
| rs569229126 | *90 |
| rs78482768 | *28 |
| rs5030655 | *6 |
| rs28371710 | *12,*45,*46 |
| rs267608302 | *50,*104 |
| rs1135824 | *3,*103,*121 |
| rs1135825 | *121 |
| rs1135826 | *121 |
| rs5030865 | *8,*14,*114 |
| rs3892097 | *4 |
| rs72549356 | *30,*40,*58,*145,*147 |
| rs745365204 | *37 |
| rs72549354 | *20 |
| rs150163869 | *20,*124,*150 |
| rs199535154 | *20,*150 |
| rs567606867 | *153 |
| rs17002853 | *131 |
| rs28371717 | *33 |
| rs72549353 | *19 |
| rs35742686 | *3 |
| rs148769737 | *84 |
| rs367543000 | *81 |
| rs72549352 | *21 |
| rs72549351 | *38 |
| rs77913725 | *81,*86 |
| rs1135828 | *81,*86,*176 |
| rs5030656 | *9,*109,*115 |
| rs1135829 | *115,*132 |
| rs267608279 | *100, |
| rs16947 | *2,*4,*8,*11,*12,*14,*17,*19,*20,*21,*28,*29,*30,*31,*32,*34,*35,*40,*41,*42,*45,*46,*51,*55,*56,*58,59,*65,**69,*73,*84,*85,*91,*98,*102,*103,*104,*105,*111,*114,*117,*121,*123,*125,*126,*128,*129,*133,*135,*136,*138,*141,*146,*148,*149,*150,*154,*155,*156,*157,*158,*159,*160,*161,*162,*163,*165,*166,*171,*172,*175,*179,*182,*183,*184 |
| rs949717872 | *24 |
| rs1406719554 | *123 |
| rs730882170 | *101 |
| rs5030867 | *7 |
| rs79292917 | *59 |
| rs72549349 | *44 |
| rs28371725 | *32,*41,*69,*91,*119,*123,*138,*158 |
| rs141009491 | *116,*174 |
| rs72549348 | *51 |
| rs78209835 | *117 |
| rs748712690 | *94 |
| rs59421388 | *29, *70,*109,*149,*155,**156,*157,*165,*171,*175 |
| rs267608295 | *25 |
| rs72549347 | *56 |
| rs76088846 | *134 |
| rs61736517 | *108 |
| rs202102799 | *108,*127 |
| rs72549346 | *42 |
| rs1555888910 | *105 |
| rs77312092 | *95 |
| rs747089665 | *135 |
| rs28371733 | *52,*106 |
| rs763964554 | *96 |
| rs3021084 | *136,*170 |
| rs569439709 | *113 |
| rs267608319 | *31 |
| rs730882251 | *62 |
| rs532668079 | *75,*154 |
| rs751092905 | *110 |
| rs369177208 | *125 |
| rs1135832 | *4,*36,*83,*141 |
| rs765776661 | *18 |
| rs1135835 | *4,*36,*83,*141 |
| rs1135836 | *4,*36,*83,*141 |
| rs141756339 | *138 |
| rs28371735 | *4,*36,*83,*141 |
| rs766507177 | *4,*36,*83,*141 |
| rs74478221 | *4,*36,*83,*141 |
| rs75467367 | *4,*36,*83,*141 |
| rs747998333 | *4,*36,*83,*141 |
| rs28371736 | *4,*36,*83,*141 |
| rs568495591 | *112 |

## Supplement 3

**Supplement 3**: CYP2D6 Inhibitor intake in a subset of the ARIANA-study („All-comer“ Registry for ImmunocArdiology aNd cardiometabolic disease Aachen) comprising patients with detected metoprolol levels (N=47).

| **Drug Name** | **Number of patients** | **Flockhart classification** |
| --- | --- | --- |
| Amiodarone | 7 | Weak inhibitor |
| (Es)citalopram | 1 | Weak inhibitor |
| Terbinafine | 1 | Moderate inhibitor |

## Supplement 4


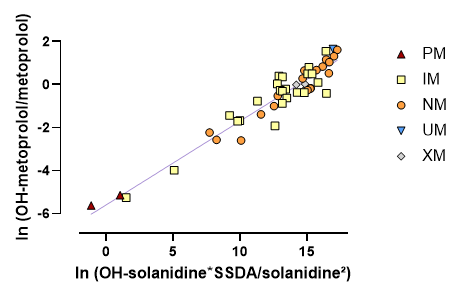


**Supplement 4:** Linear regression of the ln(OH-metoprolol/metoprolol) in a subset of the ARIANA study („All-comer“ Registry for ImmunocArdiology aNd cardiometabolic disease Aachen) comprising patients with detected metoprolol levels (N=47) with ln

𝑂𝐻−𝑠𝑜𝑙𝑎𝑛𝑖𝑑𝑖𝑛𝑒∗𝑆𝑆𝐷𝐴𝑠𝑜𝑙𝑎𝑛𝑖𝑑𝑖𝑛𝑒2*OH−solanidine∗SSDAsolanidine2*

, r=0.957 (95CI: 0.924 to 0.976), R^2^=0.916, p<0.0001.

Ln: Natural logarithm, MR: metabolic ratio, PM: genotype-predicted poor metabolizer, IM: genotype-predicted intermediate metabolizer, NM: genotype-predicted normal metabolizer, UM: genotype-predicted ultra rapid metabolizer, XM: genotype-predicted indeterminate metabolizer.Supplement 5


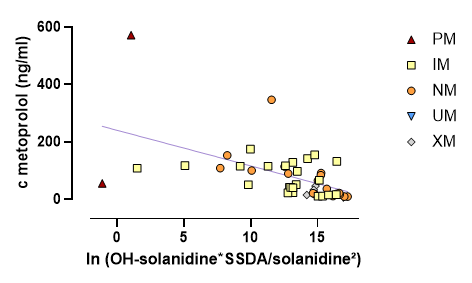


**Supplement 5:** Linear regression of metoprolol trough levels in a subset of the ARIANA study („All-comer“ Registry for ImmunocArdiology aNd cardiometabolic disease Aachen) comprising patients with detected metoprolol levels (N=47) with ln

𝑂𝐻−𝑠𝑜𝑙𝑎𝑛𝑖𝑑𝑖𝑛𝑒∗𝑆𝑆𝐷𝐴𝑠𝑜𝑙𝑎𝑛𝑖𝑑𝑖𝑛𝑒2*OH−solanidine∗SSDAsolanidine2*

, r= -0.540 (95CI: -0.716 to -0.300), R^2^=0.292, p<0.0001.

Ln: Natural logarithm, MR: metabolic ratio,, , PM: genotype-predicted poor metabolizer, IM: genotype-predicted intermediate metabolizer, NM: genotype-predicted normal metabolizer, UM: genotype-predicted ultra rapid metabolizer, XM: genotype-predicted indeterminate metabolizer, CYP2D6: cytochrome P450 2D6.
